# Nitric Oxide Transport in Normal Human Thoracic Aorta: Effects of Hemodynamics and Nitric Oxide Scavengers

**DOI:** 10.1371/journal.pone.0112395

**Published:** 2014-11-18

**Authors:** Xiao Liu, Zhenze Wang, Ping Zhao, Zhanming Fan, Anqiang Sun, Fan Zhan, Yubo Fan, Xiaoyan Deng

**Affiliations:** 1 Key Laboratory for Biomechanics and Mechanobiology of the Ministry of Education, School of Biological Science and Medical Engineering, Beihang University, Beijing, China; 2 Radiologic Department, Beijing Anzhen Hospital, Capital Medical University, Beijing, China; Albany Medical College, United States of America

## Abstract

Despite the crucial role of nitric oxide (NO) in the homeostasis of the vasculature, little quantitative information exists concerning NO transport and distribution in medium and large-sized arteries where atherosclerosis and aneurysm occur and hemodynamics is complex. We hypothesized that local hemodynamics in arteries may govern NO transport and affect the distribution of NO in the arteries, hence playing an important role in the localization of vascular diseases. To substantiate this hypothesis, we presented a lumen/wall model of the human aorta based on its MRI images to simulate the production, transport and consumption of NO in the arterial lumen and within the aortic wall. The results demonstrated that the distribution of NO in the aorta was quite uneven with remarkably reduced NO bioavailability in regions of disturbed flow, and local hemodynamics could affect NO distribution mainly via flow dependent NO production rate of endothelium. In addition, erythrocytes in the blood could moderately modulate NO concentration in the aorta, especially at the endothelial surface. However, the reaction of NO within the wall could only slightly affect NO concentration on the luminal surface, but strongly reduce NO concentration within the aortic wall. A strong positive correlation was revealed between wall shear stress and NO concentration, which was affected by local hemodynamics and NO reaction rate. In conclusion, the distribution of NO in the aorta may be determined by local hemodynamics and modulated differently by NO scavengers in the lumen and within the wall.

## Introduction

Nitric oxide (NO) secreted by vascular endothelium is a pivotal regulator in vascular homeostasis, and the change in its bioavailability has been proposed as a major mechanism of endothelial dysfunction and a crucial modulator of vascular diseases such as atherosclerosis and arterial aneurysm [1], [2]. The hallmark of endothelial dysfunction is the impaired shear stress-dependent vasodilation mediated by NO [1]. In addition to causing smooth muscle relaxation, endothelial NO possesses many cardiovascular protective functions [3]. It can reduce endothelial permeability and thus suppress the influx of lipoproteins into the vascular wall. Besides, NO can also inhibit the oxidation of low density lipoproteins, prevent leukocytes from adhesion to the endothelium and hence migration into the vascular wall, and inhibit the proliferation/migration of vascular smooth muscle cells. Moreover, NO may inhibit platelet aggregation and adhesion to the vascular walls, hence preventing thrombosis.

To perform these versatile physiological functions, NO must be transported from the site of synthesis to its target, which is a complex process in the vasculature. The major NO-producing source in the vascular system is considered to be located at the vascular endothelium and its production rate is modulated by wall shear stress (the frictional force induced by the movement of blood on the endothelial cells) [4], [5]. Once produced, endothelium-derived NO can be transported in two directions: one towards the lumen of the blood vessel and the other towards the vascular wall. In the lumen of the blood vessel, NO would be transported with the blood flow through convection and be consumed and degraded by numerous reactions, such as oxidation into nitrite, reaction with thiol group containing biomolecules to produce S-nitrosothiols, and formation of a complex with hemoglobin (Hb) [6]. Among these reactions, the most important one is the consumption by Hb that has a very high reaction rate. In the vascular wall, NO diffuses to its primary target, the adjacent smooth muscle cells, to modulate smooth muscle relaxation and vasodilation. In addition, NO in the vascular wall would also react with hemoproteins such as hemoglobin, myoglobin and cytoglobin [7], [8]. Especially, it was demonstrated that cytoglobins expressed in the fibroblasts and smooth muscle cells in the vascular wall would contribute to NO dioxygenation and behave as an important NO sink [8].

To model and analyze NO transport and reaction in the vascular system, a number of theoretical studies have been performed. However, most of the studies were focused on arterioles where atherosclerosis and aneurysm seldom develop [9]–[11]. Until now, little quantitative information exists concerning NO transport and distribution in medium and large-sized arteries where atherosclerosis and arterial aneurysm occur. Different from the arterioles, blood flow rate in the arteries is relatively high. Therefore, the convection term of NO transport may play an important role. Fadel et al [12] and Plata et al [13] theoretically investigated the production and transport of endothelial NO using a parallel plate flow model and found that the convective transport of NO had an important impact on NO distribution. Very different from the simple flow patterns in the arterioles, the blood flow patterns in the vascular disease regions such as the inner walls of curved arterial segments and the outer walls of arterial bifurcations are much more complex, and possesses flow separation and recirculation with sharp spatial variations in wall shear stress [14], [15]. Therefore, it is obvious that the complex hemodynamics in the arteries may affect the transport of NO and lead to non-uniform distribution of NO on the endothelium and in the arterial wall. We therefore hypothesized that locally impaired transport of NO and the non-uniformly distributed NO concentration may play an important role in the initialization and development of the localized vascular diseases such as atherosclerosis and arterial aneurysm. To verify this hypothesis, we numerically simulated the blood flow and the transport of NO in a lumen/wall model of a human aorta constructed based on MRI images under different hemodynamic conditions. In the numerical study, NO production rate at the blood-vessel wall interface was assumed to depend on wall shear stress and the effects of NO consumption rate in both arterial lumen and wall on NO concentration distribution were investigated.

## Methods

### Ethics Statement

The volunteer gave written, informed consent to this study that approved by the Ethical Committee of Peking University First Hospital and carried out in accordance with the regulation of the hospital.

### MR imaging and 3-D geometrical reconstruction

The geometrical model of the aortic lumen was created from images acquired from a healthy male volunteer, which was described in detail previously [16], [17]. The aortic wall was created with the thickness of the three supra-aortic vessels and thoracic aorta as 0.8 mm and 1.2 mm, respectively [18].

### Numerical approaches

#### Governing equations

*Fluid Dynamics*

Due to the very low transmural fluid velocity and high NO diffusivity in the arterial wall, the convective term of NO can be neglected [19]. Therefore, the aortic wall was assumed as solid wall without fluid transport and so that flow simulation was only carried out in the aortic lumen.

- Lumen

The flow simulation in the aortic lumen was based on the three-dimensional incompressible Navier-Stokes and continuity equations:
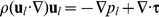
(1)

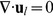
(2)where 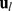 and *p_l_* represent, respectively, the fluid velocity vector and the pressure. *ρ* is the density of blood (*ρ = *1050 *kg/m* ^3^). 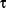 is the stress tensor [20]

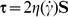
(3)where **S** and 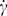 are the rate of deformation tensor and the shear rate, respectively. 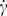 is related to the second invariant of **S**. 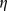 represents the viscosity of the blood, which is a function of 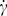. For the non-Newtonian blood flow simulation, the Carreau model is used to calculate the blood viscosity
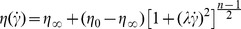
(4)where *η_∞_* = 3.45×10^−3 ^kg/(m s), *η_0_* = 5.6×10^−2 ^kg/(m s), *n* = 0.3568, 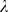 = 3.313 s [20].

*Solute Dynamics*

b. Lumen

The mass transport of NO in the flowing blood can be described by the following steady advection-diffusion-reaction equation
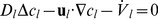
(5)where *c_l_* is the concentration of NO, *D_l_* is the diffusion coefficient of NO in blood and taken as 3.3×10^−9^ m^2^s^−1^ and 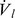 denotes NO consumed by reactions [10]. In the present study, the reaction term includes the oxidation by oxygen and the consumption by erythrocytes which are described by pseudo-second-order and first order reaction, respectively.
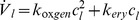
(6)where *k_oxygen_ is the pseudo-second-order reaction rate (7.56×10^−6 ^nM^−1 ^s^−1^) [13]. k_ery_ is the first order reaction rate for NO consumption by RBCs in the lumen. k_ery_ was taken to be 2.3 s^−1^ to 230 s^−1^, which was calculated from the effective second-order rate constant between NO and the RBC-encapsulated hemoglobin (from 10^3 ^M^−1 ^s^−1^ to 10^5 ^M^−1 ^s^−1^) [21],[22], with the assumption that the hemoglobin concentration in blood was 2.3 mM [23]. For a reference model, k_ery_ was taken to be 23 s^−1^.*

c. Endothelium

The NO production rate at the endothelium (*R_NO_*) is a critical parameter in the computational simulation of NO transport. A number of experimental studies measured the oxidation products of NO and estimated the NO production rate at the endothelium [11], [24]. However, due to the differences in experimental protocols, the reported data varied in a very wide range (from 0.035 to 68 µM/s) [11], [24]. To investigate the effect of wall shear stress dependent NO production rate on the transport of NO, two separate models were compared. The reference one is the hyperbolic model which is based on the experimental measurements by Andrews et al [25] as described by:
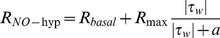
(7)where R*_basal_* = 2.13 nMs^−1^, R*_max_* = 457.5 nMs^−1^ and *a* = 3.5 Pa.

The second is assumed to have a R_NO_ that was linearly dependent on WSS as described by [26]:
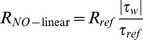
(8)where *R_ref_* = 150 µM s^−1^, *τ_ref_* = 2.4 Pa.

d. Arterial wall

The transport of NO in the arterial wall was modeled by the following equation
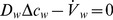
(9)where *c_w_* is the concentration of NO in the arterial wall, *D_w_* is the diffusivity of NO in arterial wall (8.48×10^−10^ m^2^s^−1^) and 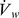 is the reaction of NO [27]. The reaction is treated as a first-order rate expression, which is given by [28]

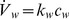
(10)where *k_w_ is the consumption rate constant, which was assumed to be 0.01 s^−1^ and 0.1 s^−1^, respectively to investigate the role of wall consumption on the NO transport and distribution [28]. For the reference model, k_w_ was assumed as 0.01 s^−1^.*

#### Boundary conditions

Flow transport equations (Eqs 1∼2) are subject to the following boundary conditions:

BC-A: For the reference model, at each of the 3 aortic arch branches, 5% of flow volume was allowed to be ejected [17]. Studies demonstrated that the flow volume division at the outlets of the aorta can significantly affect hemodynamics in the aorta [29]. To investigate the effect of different flow volume division on NO transport, we also carried out simulations with flow volume at brachiocephalic artery, left common carotid artery and left subclavian artery as 13.4%, 10.6% and 12.0%, respectively [29].

BC-B: For the reference model, a flat inlet flow velocity profile was used assuming the time-average *Re* = 790 (velocity is 0.1 *m/s*) [17]. Blood flow rate (hence, Re) in the aorta varies dramatically from a resting state to a state of a strenuous exercise. Therefore, to verify the effect of hemodynamics on NO transport, we also carried out simulations at Re = 1185(one and a half of the reference one) and 1580 (twice of the reference one).

The boundary conditions for the mass transport equations (Eqs (5) and (9)) are as follows:

*BC-1*: The inflow concentration of NO (*c_0_*) at the inlet is assumed to be 0 nM.
*BC-2*: The continuity of NO concentration is maintained and the mass flux across the endothelium is assumed to be the product of the endothelium NO production rate and the thickness of the endothelium. That is

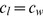
(11)

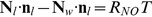
(12)

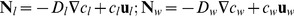
(13)where 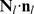 is the flux of NO from the endothelium into the arterial wall, 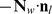 is the flux of NO from the endothelium into the lumen, *T* is the thickness of endothelium, which is assumed to be 2 µm and *R_NO_* is the NO production rate.

*BC-3*: For other boundaries, the concentration gradient in the boundary normal direction is assumed to be zero.

#### Mesh grid

**Figure 1:**
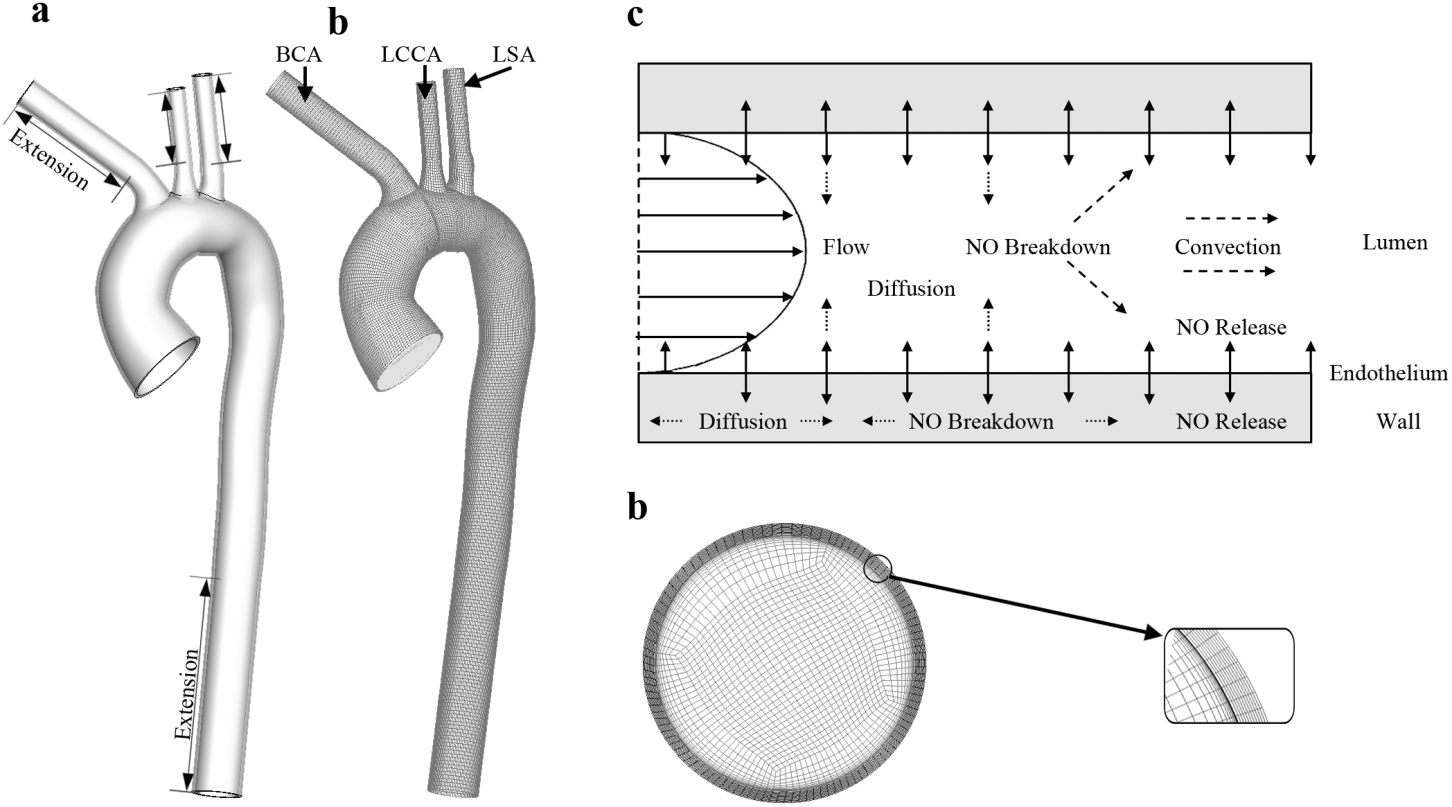
Illustration of the computational models. (a) Geometrical model of the investigated thoracic aorta with straight flow extensions to minimize the influence from outlet boundary conditions. (b) Computational meshes of the thoracic aorta. A structured hexahedral mesh was used with high quality hexahedral cells near the wall to capture the steep variation in NO transport within a thin fluid layer adjacent to the vessel wall. BCA (brachiocephalic artery), LCCA (left common carotid artery), LSA (left subclavian artery). (c) Schematic representation of the mathematical model. Wall shear stress dependent NO release at the endothelium was transported into arterial lumen and wall where physical (diffusion and convection), and biological (consumption) processes were undertaken.

A hexahedral mesh was produced using blocking and O-grid techniques with high quality hexahedral cells near the wall to capture the steep variation in mass transport within a thin fluid layer adjacent to the vessel wall (Fig. 1). In order to assure that the results were mesh-independent, the grid-adaptation technique was used, which refined the grid based on the geometric and numerical solution data. Mesh independence was considered to be achieved when the averaged difference in NO concentration between two successive simulations was less than 4%. Mesh independence was reached at 402,556 hexahedral cells for aortic wall and 813,060 hexahedral cells for aortic lumen, respectively.

#### Computation procedures

The numerical calculations were carried out using a validated finite volume-based algorithm Fluent (Ansys. Inc. USA) with a user-defined C-like function (UDF). The UDF was used to solve the mass transport equations and validated by the results from Liu et al [19].

#### Statistical Analysis

To quantitatively investigate the correlation between WSS and NO concentration at the endothelium, we computed the Spearman rank correlation coefficient (*r*) between them for all the simulation cases using MATLAB (The MathWorks, Natick, MA, USA).

## Results

### Hemodynamics may govern the non-uniform NO distribution in the aorta

**Figure 2:**
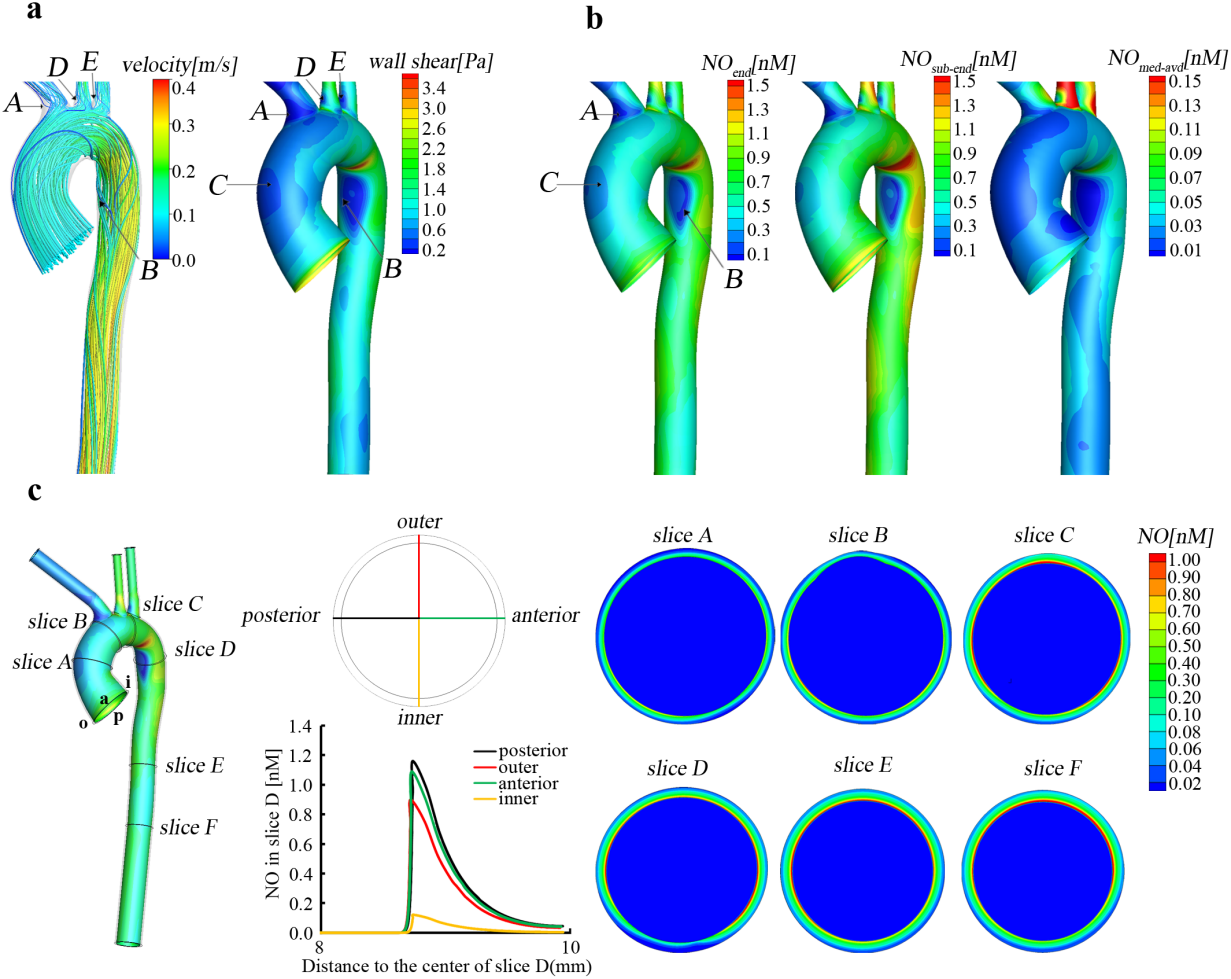
Uneven distributions of wall shear stress and NO in the aorta. (a) The streamline shows a swirling flow pattern and the wall shear stress is relatively low in the disturbed flow regions (A, B, D, and E). (b) The distributions of NO concentration at the endothelial surface towards lumen (*NO_end_*) and wall (*NO_sub-end_*), and at the media/adventitia interface (*NO_med-adv_*) are very uneven and relatively low in the disturbed flow regions. (c) NO distributions of 6 representative slices in the aorta are used to show the NO distributions in the aortic lumen and wall. The model is with a contour plot of *NO_end_*. a, p, o, i marked on the model is anterior, posterior, outer and inner wall of the aorta. The distributions of NO at slice D in the four directions is shown quite uneven.

Figure 2a shows the streamline and the distribution of wall shear stress (WSS) of the reference model. As shown in the figure with streamline, the blood flow takes a form of swirling flow from the ascending aorta to the aortic arch and separates from the mainstream forming disturbed flow patterns at the entry area of the three supra-aortic vessels (Regions A, D, E) and the distal end of the aortic arch (Region B). In these disturbed flow regions, WSS is relatively low. Particularly, the lowest WSS areas are located at regions A and B. Another low WSS region is located at the ascending aorta (Region C).

Figure 2b illustrates NO distribution at the lumen/wall interface and at the media/adventitia interface (*NO_med-adv_*). At the lumen/wall interface, NO distributions at both the lumen-side and wall-side of the endothelium are analyzed. The luminal surface concentration (*NO_end_*) characterizes the effects of NO convection and consumption in the lumen while the wall-side concentration (*NO_sub-end_*) provides information of NO bioavailability in the subendothelial layer. As shown in the figure, the distribution of *NO_end_* is quite similar to that of WSS, which is very uneven in the aorta. In the ascending aorta *NO_end_* along the inner wall is relatively lower than that along the outer wall. In contrast, in the descending aorta *NO_end_* along the inner wall is relatively higher. Similar to WSS, *NO_end_* at disturbed flow regions A and B is the lowest. When compared to *NO_sub-end_*, *NO_end_* is relatively lower due to the convection and consumption in the lumen, but their distributions strongly resemble. The consumption reaction in the aortic wall may significantly reduce NO bioavailability, leading to *NO_med-adv_* more than ten times lower than *NO_sub-end_*.

To better present NO transport process across the aortic wall, NO distributions in six representative slices along the longitudinal direction of the centerline of aorta are shown in figure 2c. It is clear that NO concentration in the aorta wall is non-uniform and it is relatively low in the inner wall. Specifically, NO concentration in the inner wall of the distal end of the aortic arch (slice D) is almost ten times lower than that in the outer wall.

**Figure 3:**
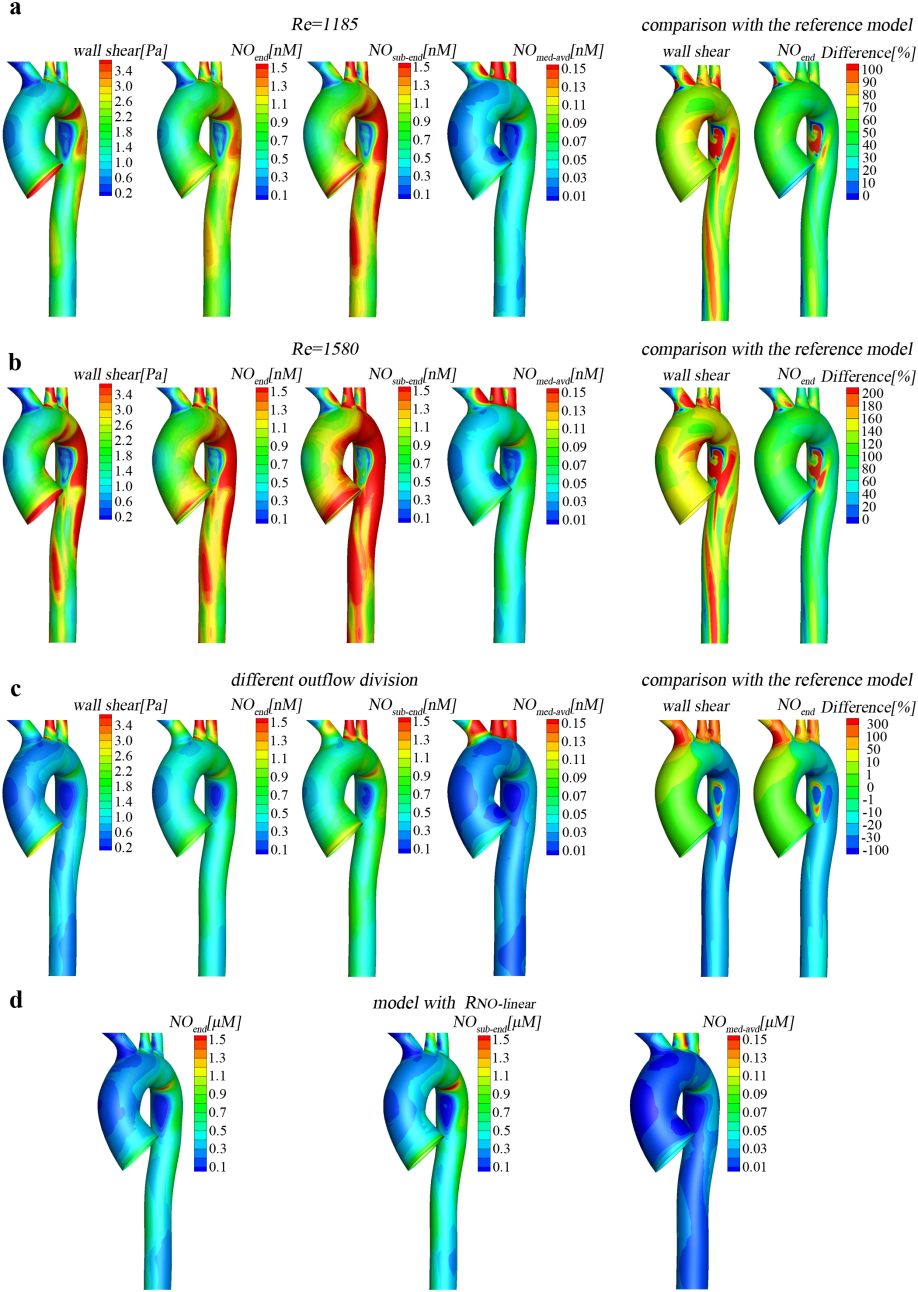
Changes in hemodynamics and shear dependent NO production rate affect the NO distribution in the aorta. (a–b) Comparison of the WSS and NO distribution between the reference simulation and the one at Re = 1185(one and a half of the reference one) and the one at Re = 1580 (twice of the reference one). Percentage difference = (*NO−NO_reference_*)/*NO_reference_*_._ (c) Effects of different flow volume division at the outlets of the aorta. (d) The distributions of NO concentration for the NO production release model of R*_NO-linear_*.

As shown in Fig. 3a–b, generally speaking, the increase in Re causes an increase in both WSS and NO concentration, and the increase in the latter is according with that in the former from place to place. However, the increase rate of WSS is more than that of NO concentration, especially in the inner wall of the aorta. Moreover, as shown on figure 3, the increase rate of WSS and NO is quite uneven in the aorta. For instance, when compared to the reference model, WSS and *NO_end_* for Re = 1185 increases approximate 50% in most regions of the aorta, but more than 100% at region D.

As demonstrated in Fig. 3c, when compared to the reference model, both WSS and NO is significantly reduced in the descending aorta due to the decrease in flow volume and greatly increases in the three supra-aortic vessels. In addition, the differences in NO concentration and WSS between the present model and the reference one are closely similar, which indicates that hemodynamics in the lumen of aorta can directly affect NO distribution in the aorta.

It is well documented that the NO production rate of the vascular endothelium depends on hemodynamics, specifically wall shear stress. We therefore calculated NO transport with other product rate that is linearly dependent on WSS (Eq. (8) in the section of method). As demonstrated in Fig. 3d, when compared to the reference simulation based on *R_NO-hyp_* in Eq. (7), due to the much higher NO production rate, NO concentration obtained for *R_NO-linear_* is much higher. For the two models, as illustrated in Fig. 2(b) and Fig. 3d, NO concentration is relatively low at regions A, B, C. However, close comparison finds that the distributions of *NO_end_* and *NO_sub-end_* with *R_NO-linear_* are more similar to the distribution of WSS than those with *R_NO-hyp_*. These results indicate that the magnitude and the form of NO production model have a great influence on the concentration distribution of NO in the aorta.

### The consumption rate of NO in the lumen modulates NO distribution in the aorta

**Figure 4:**
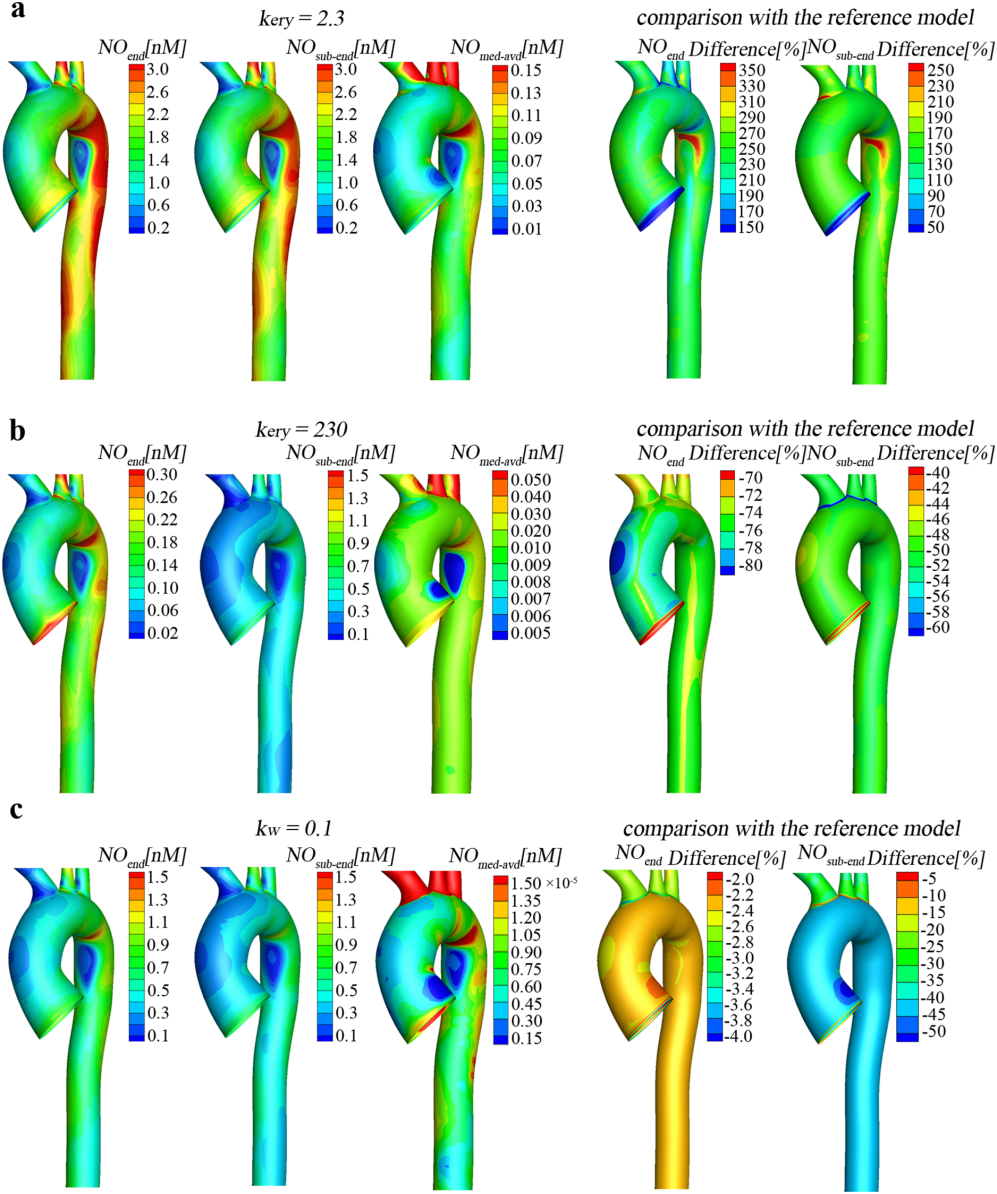
Effect of NO scavengers on the NO transport. The consumption rate of NO by erythrocytes (*k_ery_*) in the lumen modulates NO distributions in the aorta. (a–b) Comparison of the NO distribution between the reference simulation and the one at *k_ery_* = 2.3 (one tenth of the reference one) and at *k_ery_* = 230 (ten times of the reference one). (c) Effects of the consumption rate of NO in the aortic wall *k_w_* = 0.1 (ten times of the reference one). *k_w_* strongly impacts the NO concentration in the aortic wall but slightly affects that at the surface of the endothelium to the lumen (*NO_end_*).

NO transported to the arterial lumen would be consumed by numerous reactions, especially by hemoglobin in erythrocytes at very high rate. To investigate the effects of the consumption rate of NO in the lumen on NO transport, simulations were carried out for several values of NO consumption rate by erythrocytes (*k_ery_*). As is evident from Fig. 4a–b, the distribution of NO concentration in the aorta is very uneven for different *k_ery_* and in all the cases the transport of NO is significantly hindered in the disturbed flow regions (Regions A and B), which further indicates hemodynamics plays an important role in the distribution of NO in the aorta. In addition, as shown in Fig. 4a–b, as *k_ery_* increases, NO concentration at both the lumen/wall interface and the media/adventitia interface reduce remarkably, indicating that erythrocyte is an effective NO scavenger in the blood that modulates NO concentration in the arterial lumen and wall. More specifically, *k_ery_* may have a more significant influence on NO concentration at the endothelial surface than in the arterial wall. When compared to the reference model with *k_ery_* = 23 s^−1^, for the case with *k_ery_* = 2.3 s^−1^, NO concentration increases approximately 250% at the endothelial surface (*NO_end_*) but only about 150% at the sub endothelial region (*NO_sub-end_*) and the media/adventitia interface (*NO_med-adv_*), and *NO_end_* is a bit higher than *NO_sub-end_*. However, for the case with *k_ery_* = 230 s^−1^, *NO_end_* decreases sharply approximately 75%, and quite lower than *NO_sub-end_* that decreases only 45%.

### The reaction of NO in the wall strongly affects NO concentration in the aortic wall

NO diffusing into the vascular wall would be scavenged by smooth muscle cells and react with some hemoproteins. The measured reaction rate (*k_w_*) is quite different and affected by the physiological condition [30]. To investigate the effects of consumption in the arterial wall on NO transport and distribution, simulations with *k_w_* = 0.1 s^−1^ were carried out and compared with the reference model with *k_w_* = 0.01 s^−1^. As shown in the Figure 4c, the increase in *k_w_* has almost no effect on *NO_end_* but greatly reduces NO concentration in the aortic wall (*NO_sub-end_* and *NO_med-adv_*). For almost all the area of the aorta, the percentage difference in *NO_end_* is only about 2%. However, the percentage difference in *NO_sub-end_* is about 40% at the thoracic aorta and approximately 20% at the three supra-aortic vessels. The increase in *k_w_* decreases *NO_med-adv_* almost three orders of magnitude. These results indicate that the reaction of NO in the wall has strong effects on NO concentration in the aortic wall but no effects on the NO at the endothelial surface of towards the lumen.

### Positive correlation between WSS and NO concentration at lumen/wall interface

**Figure 5:**
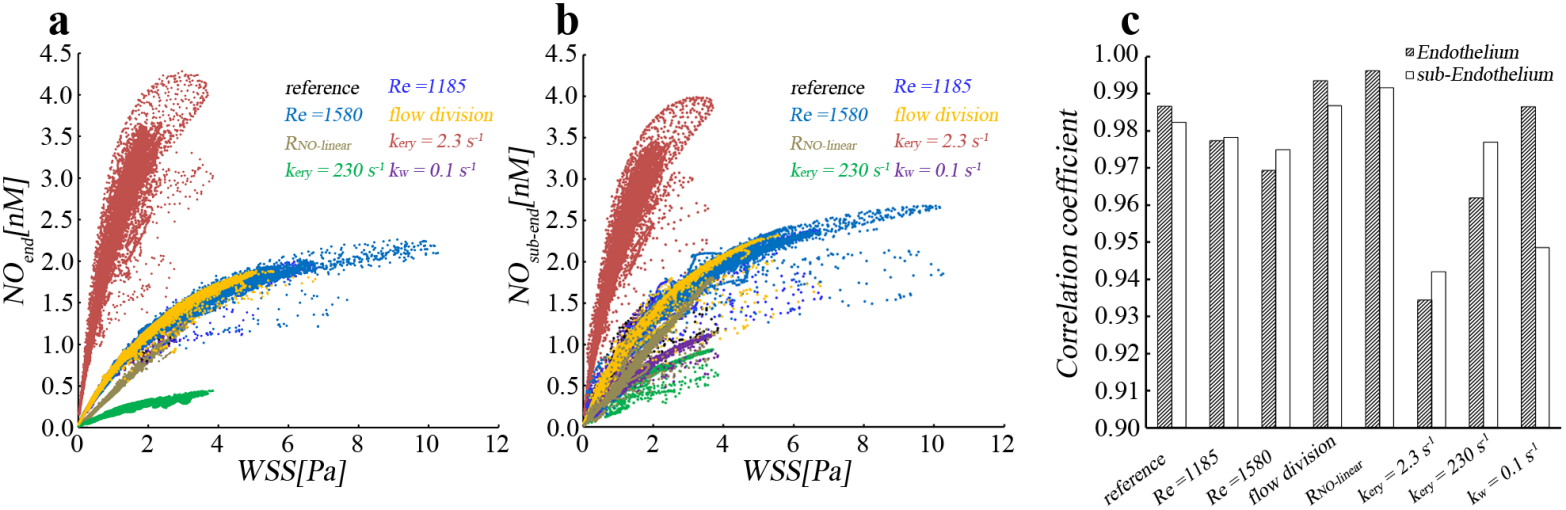
Positive correlation between wall shear stress (WSS) and NO concentration. (a–b) The relationship between WSS and NO concentration at the endothelial surface towards lumen (*NO_end_*) and wall (*NO_sub-end_*). The unit of NO concentration for R*_NO-linear_* is µM. (c) The spearman rank correlation coefficient between WSS and NO concentration.

Figures 5a–b show the relationship between wall shear stress and NO concentration at both the lumen-side and wall-side of the endothelium under different hemodynamics and reaction conditions. The figures were constructed by plotting the value of NO concentration against that of wall shear stress at each mesh node of the lumen/wall interface. It is evident that there is a relationship between WSS and NO concentration and NO concentration increases with increasing WSS. When WSS values are reduced to zero, NO concentration is close to zero. The results also demonstrate that profiles of the relationship between NO concentration and WSS for cases with Re = 1185, Re = 1580 and different flow volume division at outlets are overlapped, which indicates that the profile is almost independent on hemodynamics for the same NO production rate model. However, the profile of the relationship is significantly modulated by the production rate of NO and the consumption rate of NO in the lumen, and that between *NO_sub-end_* and WSS is further affected by the reaction rate of NO in the wall.

Figure 5c illustrates the spearman rank correlation coefficient (*r*) between WSS and NO concentration. *r* is more than 0.93 for all the simulation cases, which indicates that a strong positive correlation exists between WSS and NO concentration. Moreover, the figure also shows that the correlation coefficient is affected by both hemodynamics and NO reaction rate in both arterial lumen and wall. For instance, when Reynolds number increases, *r* for both *NO_end_* and *NO_sub-end_* may decrease and *r* becomes smaller for *NO_end_* than for *NO_sub-end_*. In addition, when *k_ery_* is too low or too high, the consumption rate of NO in the lumen (*k_ery_*) may decrease *r*. The reaction rate of NO in the wall may not affect *r* for *NO_end_* but may greatly reduce *r* for *NO_sub-end_*, when *r_w_* increases. These results indicate that WSS is a key factor that determines the local concentration of NO, and it is modulated by other factors, such as *k_ery_* and *k_w_*.

## Discussion

It is well documented that nitric oxide may have numerous cardiovascular protective properties [1], [3]. In the present study, we hypothesized that hemodynamics in the arteries may govern NO transport and lead to non-uniform distribution of NO in the arteries, hence playing an important role in the localization of vascular diseases such as atherosclerosis and arterial aneurysm. To substantiate this hypothesis, we numerically simulated the blood flow and NO transport in a lumen/wall model of a human aorta. Our results demonstrated that NO distributed unevenly in the aorta and reduced remarkably in disturbed flow regions (Fig. 2). By setting different flow boundary conditions such as the inlet blood flow rate (hence Re) and the outlet flow rate division, NO distribution in the aorta may be altered significantly (Fig. 3), indicating that alterations in hemodynamics such as exercise or stenosis may directly affect NO transport. Since NO production rate is WSS-dependent, hemodynamics may affect NO distribution mainly via shear dependent product rate (Fig. 3). Moreover, the erythrocyte in the lumen was an effective NO scavenger that modulated NO concentration in both the arterial lumen and wall, especially at the endothelial surface towards the lumen (Fig. 4). However, the reaction of NO in the wall had little effects on the luminal NO concentration, but could significantly affect NO concentration within the aortic wall (Fig. 4). The statistical analysis demonstrated that a strong positive correlation existed between WSS and NO concentration, and was affected by hemodynamics and NO reaction rate (Fig. 5).

Similar to the previous findings in the aorta, blood flow takes a form of swirling flow, but in some particular regions flow disturbance occurs [29], [31]. Our results showed that in these disturbed flow regions, NO concentration on the endothelium and within the arterial wall were significantly reduced. For instance, NO concentration in the inner wall of the distal end of the aortic arch where flow disturbance occurs was almost ten times lower than that in the outer wall. The reduced NO bioavailability was largely due to the low NO production rate because of low WSS there. Several experimental studies demonstrated that endothelial nitric oxide synthase (eNOS) in the atheroma-prone regions with flow disturbance was either absent or significantly lower than that in normal flow regions [32],[33]. It has been documented that when compared with those atherosclerosis-free regions, the atherosclerotic segments of the arteries has a remarkably reduced NO release production rate [34]. The reduced NO concentration in the disturbed flow regions might lead to atherogenesis by enhancing leucocyte adhesion to the endothelium and modifying low-density lipoproteins in the arterial wall [35], [36]. In addition, mice model studies demonstrated that the deficiency in endothelial nitric oxide synthase would accelerate the development of atherosclerosis and induce spontaneous aortic aneurysm and dissection [37].

The present study investigated the effect of NO production rate on NO transport using two different models, one is hyperbolic model, and the other is linear model. The former may be more physiological than the latter, since NO concentration on the endothelium obtained with the former is several nM, which is the same order of magnitude measured on the endothelial cells and in dog coronary sinus [38], [39]. Moreover, the results obtained revealed that the magnitude and the form of NO production rate have a great influence on the concentration distribution of NO in the aorta, indicating that abnormal NO production rate of dysfunctional endothelial cells may lead to the development of atherosclerosis. This theoretical finding may give an explanation to the experimental findings by others [40], [41]. For instance, animal model studies demonstrated that increase in NO production by treatment with L-arginine, the precursor of nitric oxide, inhibited atherosclerotic lesion formation [40]. On the contrary, inhibition of NO production through L-arginine analogues such L-nitroarginine methylester (L-NAME) could significantly accelerate atherosclerotic lesion development [41].

Nitric oxide released from endothelial cells into blood reacts with several molecules. The reaction of NO with hemoglobin, which has a rapid reaction rate, accounts for several pathologies such as pulmonary and systemic hypertension, sickle-cell disease [42]. Our results showed that that ten time increase in NO consumption rate by erythrocytes only leads to 75% decrease in NO concentration on the endothelium and less than 50% decrease within the aortic wall, indicating that erythrocyte may not be a very strong modulator of NO in the aorta and it affects NO bioavailability on the endothelial surface more than that within the arterial wall. This phenomenon is different from the findings observed on the arterioles, in which hemoglobin is shown to be a strong NO scavenger [11]. The discrepancy might mostly be attributed to the relatively high blood flow velocity in the aorta, which may lead to a very thin concentration boundary layer near the aortic wall (see Figure 2c) and hence reduce the regulatory effect of the hemoglobin. The results may partly explain the phenomenon that atherosclerosis does not occur more preferentially in subjects with sickle-cell disease [43].

NO diffusing into the adjacent smooth muscle cells activates soluble guanylate cyclase (sGC), which catalyzes the formation of cyclic guanosine monophosphate (cGMP), leading to smooth muscle relaxation and vasodilation. It has been reported that NO concentration required to activate sGC varies in a wide range (100 pM–5 nM) [30]. The present study demonstrated that if *k_w_* (the NO consumption rate constant in the arterial wall) was assumed to be 0.01 s^−1^, the predicted NO concentration may be sufficiently high to activate sGC. However, if *k_w_* increased ten times, NO concentration in the wall may decrease by approximately three orders of magnitude so that unable to activate sGC. These results indicate that the consumption rate of NO in the wall may be a very important factor that regulates NO concentration in the aortic wall. In fact, both human and animal model studies have demonstrated that augmented NO scavengers such as reactive oxygen species in atherosclerotic vessels may remarkably degrade NO in the arterial wall and hence accelerate atherosclerosis [44].

Flow-induced wall shear stress (WSS) has been recognized as one of the most important haemodynamic factors in the localization of vascular diseases [2], [15]. Nevertheless, the underlying mechanism is still not very clear. WSS can modulate the biological function of the endothelium both via regulating the mechanotransduction of the endothelial cells and affecting the local concentration of chemicals/agonists at the endothelial surface [45]–[47]. As indicated in the present study, a strong positive correlation exists between WSS and NO concentration, which implies that WSS may also affect the endothelial biology through controlling NO concentration. The notion can be partly supported by some recent studies demonstrating that similar to WSS, flow-dependent mass transfer may also trigger endothelial cell signaling cascades [48].

In the present study, the blood flow was assumed steady and the compliance and motion of the aorta was neglected, which may affect the accuracy of our prediction on the absolute value of NO concentration. However, it can still shed some light on the general tendency of NO distribution in the aorta, since it is demonstrated that the compliance of the aortic wall and the pulsation of blood flow have a little effects on the distribution of WSS and exogenous macromolecules and micromolecules in the aorta [17], [19], [49]. Another limitation of the present study is that the RBC free plasma layer on the endothelial cells was not included in the modeling. Due to the much lower NO reaction rate in the RBC-free plasma layer, the influence of RBCs on NO transport in the aorta may be diminished by the existence of the layer. Therefore, the overall NO concentration predicted on the arterial wall may be underestimated. However, the NO distribution in the aorta may not be changed significantly by the simplification, since it was found that the layer had little effects on the distribution of NO in an idealized model of stenosed artery [19]. In addition, it should be pointed out that flow shear stress could regulate eNOS activity hence NO production through both transcriptional mechanism within seconds or minutes and posttranscriptional one within hours or days [50], [51]. As the flow in the arteries in vivo is pulsatile, which would lead to dramatic changes in WSS on cells in a short time, the NO production in vivo might be the combining results of the two mechanisms. Under steady flow condition, both the two mechanisms would lead to a rapid increase in NO production rate at relatively low WSS, but at relatively high WSS, the transcriptional mechanism would cause more NO production [25], [52]. The main NO production rate model (eq. 7) used in the present study was based on experimental measurements obtained within minutes, which may result from the transcriptional regulation of eNOS and hence may lead to overestimated production rate at relatively high WSS. Therefore, the distributions of NO at regions with high WSS may be somewhat overestimated. However, the NO distribution at low WSS region, which was the most concerned region, may be reasonable. Moreover, considering the great variance of NO production rate, we also calculated the NO transport using linear model (eq. 8, Figure 3d) which is almost three larger orders of magnitude than the hyperbolic model. The results of the two models were consistent, which indicated that despite the concentration value simulated might be not very accurate, the distribution would be reliable and have biological significance. To assess accurately the NO concentration value, NO production rate in response to different physiological flow conditions needs to be further measured and used in simulations.

## Conclusions

Local hemodynamics may govern NO transport in the aorta. Disturbed flow in certain areas of the aorta could remarkably reduce NO bioavailability that may play an important role in the localization of vascular diseases such as atherosclerosis and arterial aneurysm. In addition, NO bioavailability in the aorta was modulated differently by the NO scavengers in the arterial lumen and in the arterial wall. NO bioavailability in the wall was very strongly affected by NO scavengers in the arterial wall, which implied that elimination of some of the scavengers may be an efficient approach to increase NO bioavailability and thus alleviate vascular disease.
